# A Case of Midbrain and Thalamic Infarction Involving Artery of Percheron

**DOI:** 10.3390/jcm4030369

**Published:** 2015-03-03

**Authors:** Muhammad Almamun, Appu Suman, Syed Arshad, Sonni Jayathirthachar Sanjeev Kumar

**Affiliations:** 1 Department of Neurology, Salford Royal NHS Foundation Trust, Stott Lane, Salford, Manchester M6 8HD, UK; E-Mails: a.suman@doctors.org.uk (A.S.); arshad003@hotmail.com (S.A.); 2 Department of Stroke Medicine, Blackpool Teaching Hospitals NHS Foundation Trust, Whinney Heys Road, Blackpool FY3 8NR, UK; E-Mail: sanjeev.kumar@bfwhospitals.nhs.uk

**Keywords:** Artery of Percheron (AOP), stroke, mid-brain, thalamus, infarct, bilateral lesions

## Abstract

Blood supply to the thalamus and brainstem have frequent anatomic variations. One of these is where all the perforators to the above areas arise from a single branch of the posterior cerebral artery commonly known as the artery of Percheron. Infarction involving this artery leading to bilateral thalamic and midbrain lesions is not uncommon, but can cause diagnostic difficulties due to the varying clinical presentations possible and the wide differentials. Early brain imaging and diagnosis is important for initiating appropriate treatment. In this case report, we discuss a patient who presented with an artery of Percheron related stroke affecting the mid brain and paramedian thalamic areas. We also discuss the differentials of presentations with similar symptoms.

## 1. Introduction

The blood supply to the thalamus is primarily from the perforating arteries from the Posterior Communicating Artery (PCom) and the Posterior Cerebral Artery (PCA). Venous drainage takes place through the internal cerebral veins and basal veins of Rosenthal which drain over the great vein of Galen into the Straight Sinus. Gerard Percheron described multiple variations of the arterial supply in one of which all these perforators arise from a single branch [1] of the PCA—Artery of Percheron (AOP).

Varying clinical presentations are noted in patients with stroke which involves the artery of Percheron. The structures affected are associated with the regulation of alertness, consciousness and sleep. Occlusion of this artery presents with variable symptoms which mimic inflammation, infection or malignant features baffling the clinicians [2]. Studies found that AOP infarction involves around 0.1% to 0.3% of all ischaemic strokes of which 22%–35% are associated with thalamic infarction [3]. Thalamic stroke which involves the AOP has four distinctive characteristics of ischemia according to the areas involved. There is always bilateral paramedian thalamic involvement with varying involvement of midbrain and anterior thalamus [4]. Variable mental alertness with confusion and coma is invariably typical at presentation.

## 2. Case Report

A seventy-year old gentleman presented to the Accident and Emergency (A&E) department with reduced level of consciousness. His wife had heard a thud and found him collapsed and disoriented in the bathroom. She also noticed that her husband was talking to himself and moving both arms. His level of awareness dropped rapidly and became unconscious. His past medical history includes hypertension and asthma. He was an ex-smoker and drank alcohol in moderation. He had no known allergies. He was right handed. His Modified Rankin Scale score (MRS) prior to admission was zero. He had no past history of substance abuse, head injury, trauma or seizure activity.

On arrival to Accident and Emergency (A&E) his Glasgow Coma Scale (GCS) was 5/15 (Eyes 1, Verbal 1, Motor 3). On examination, pupils were unequal, left pupil pin-point, right pupil 3 mm—both unreactive to light. There was quadriparesis, bilateral up going planters, bradycardia (40–50 beat per min) with relative hypotension (Blood Pressure 95/68 mmHg). The National Institutes of Health Stroke Scale (NIHSS) score was twenty six (26). Cardiovascular, respiratory, gastrointestinal examinations were normal. Blood glucose level was 6.6 mmol/L. Patient was intubated and ventilated. CT Brain Scan did not show any evidence of acute intracranial hemorrhage. A CT Cerebral Angiogram excluded any major intracranial artery occlusion and showed evidence of bilateral thalamic hypodensity with probable hypodensity in the mid brain. The patient was started on high dose of aspirin for ischaemic stroke as per standard guidelines [5]. In view of high NIHSS score and unclear onset of stroke symptoms thrombolysis was not considered because risk of hemorrhagic transformation outweighs the benefits.

**Figure 1:**
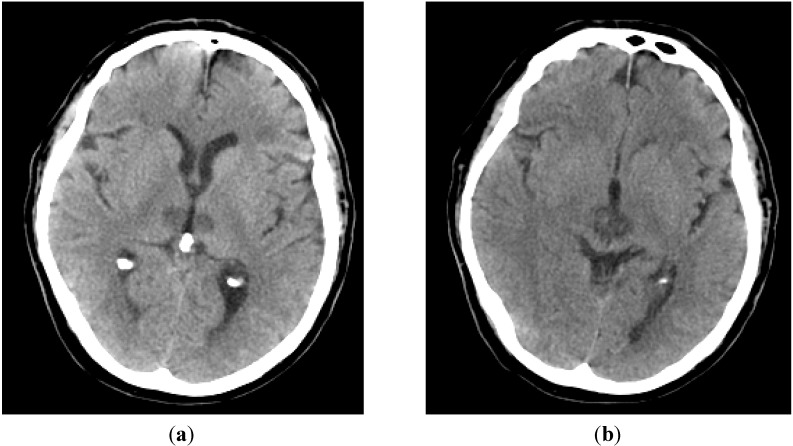
Bilateral thalamus and mid brain infarction involving Artery of Percheron. (**a**) Bilateral thalamic infarcts; (**b**) Bilateral midbrain infarcts.

The patient was extubated after the scan and was breathing spontaneously, his pupils still remained unequal. He briefly woke-up, looked around and spoke to his family. On the following day, GCS dropped to 7/15 (E2, V2, M3) again with unequal pupils—right pupil 4 mm reactive and left pupil 1 mm unreactive. There was complete ptosis of the right eye with loss of adduction and upgaze. There were no features of seizure activity in this period. He had Cheyne Stokes breathing with occasional spontaneous movement of limbs and bilateral up going planters. Investigations including Chest X-ray, Electrocardiogram and routine blood results were inconclusive. A repeat CT Brain Scan showed established bilateral thalamic and medial midbrain hypodensities consistent with an established AOP infarction (See Figure 1). His level of consciousness was fluctuating. He improved gradually with spontaneous eye opening and obeying simple commands although communication was limited to yes/no answers on recognition of voices. He developed pneumonia which was treated with antibiotics along with severe obstructive sleep apnoea requiring Continuous Positive Airway Pressure (CPAP) support. Nutrition was maintained through total parenteral nutrition (TPN). A radiologically inserted Gastrostomy (RIG) tube for continuing nutrition supplement was planned. ECG monitoring detected atrial flutter that was new this admission. Anticoagulation with treatment dose of Low Molecular Weight Heparin (Tinzaparin) was initiated. Following 12 weeks of inpatient treatment along with extensive rehabilitation the patient was discharged with a package of care to his home. At the time of discharge, his deficits were reduced attention in right side with minimal residual weakness, dysarthria, R eye gaze palsy with complete ptosis. His NIHSS score improved to six but he had impulsivity with low safety awareness, poor midline awareness, inability to maintain his position and unsteady with gait. Swallowing improved considerably to allow modified diet. On discharge, his Barthel score was two (2/20) and Modified Rankin Score was 4 requiring nursing level of care and support. Multidisciplinary teams were closely involved in terms of his therapy assessment, nursing care, nutritional support, anticoagulation and discharge package of care.

## 3. Discussion

Infarction associated with thalamus and midbrain due to occlusion of artery of Percheron is difficult to diagnose clinically due to the variability involvement of thalamus and midbrain, and ambiguous clinical presentations [6]. There are many differentials to keep in mind (Table 1) when a patient presents with bilateral lesions of the thalamus.

**Table 1:** Differentials of presentations of Artery of Percheron Infarcts [7,8].

Differentials	Acute/Subacute Presentations
Vascular–Arterial	Stroke-Infarct-tip of basilar artery syndrome, PoA occlusion
Vascular–Venous	Cerebral Vein Thrombosis especially Great vein of Galen, Straight Sinus thrombosis
Infections–Viral	Flavi virus infections e.g., Japanese Encephalitis (90% of patients have bithalamic involvement), West Nile Virus encephalitis
Infections–others	Tuberculous Meningio-encephalitis, Fungal Infection, Malaria, Toxoplasmosis
Demyelination	Acute Demyelinating Encephalomyelitis, Multiple Sclerosis
Spongiform encephalopathy	Variant Creutzfeldt-Jakob disease (vCJD), CJD
Thiamine deficiency	Wernicke’s encephalopathy
Hypoxic injury newborn	Profound Hypoxia of the Newborn
**Non-Acute Presentations**	
Vascular	Chronic Hypertensive encephalopathy leading to lacunar infarcts/microbleeds
Tumours	Astrocytoma, Glioblastoma Multiforme, Germinoma, lymphomas (1% of Central nervous System tumours)
Congenital Metabolic Syndrome	Leigh Syndrome (mitrochondropathy), Gangliosidoses (Lysosomal disorders), Krabbe’s disease, Wilson’s Disease
Cerebrovascular Ferrocalcinosis	Fahr’s Disease

In this case study, the patient had a sudden disorientation with collapse followed by unconsciousness that needed urgent medical attention. Infarction of AOP showing mid brain involvement demonstrates poor long term prognosis [9]. Our patient had varying level of consciousness following his admission initially needing intubation and later CPAP support. Three common recognized features—alteration of mental status, impairment of cognition and vertical gaze palsy frequently present which was observed in this case presentation throughout his unstable clinical condition [10]. Some cases patient may present with hemiplegia, ataxia and occulomotor disturbance suggesting bilateral thalamic infarction (thalamopeduncular syndrome) [11]. A variety of clinical signs including dysarthria, impaired convergence, retraction of eyelids, amnesia, ataxia, involuntary movement of limbs have been described [10]. In this case, ECG monitoring a few days later detected atrial flutter which we was presumed to be source of embolism of his POA stroke. Cardioembolism is one of the most common types of aetiology which occludes the AOP leads to bilateral paramedian thalamic infarction [12]. Although the CT Brain does not always confirm AOP infarcts in many scenarios, in this case it was detected early. MRI brain with diffusion-weighted imaging (DWI) would be ideal to diagnose infarction early [13] in most situations. However, many studies report normal imaging results in early CT and MR (DWI) brain scan in a symptomatic and even comatose patients with AOP ischemic infarction [14]. Patients with basilar artery syndrome with occluded Artery of Percheron can be considered for intra-arterial thrombolysis. In this case, there was a fall in the bathroom which alerted his wife, and even though this time was clear, it was not sure whether the patient already had the stroke while asleep and his attempt to walk unaided resulted in the fall. Hence he was not considered for thrombolysis. In this case, he was treated with therapeutic dose of Tinzaparin (LMWH) to cover the risk from his atrial flutter.

## 4. Conclusions

Acute onset of low conscious levels with localizing neurology to midbrain and thalamus could be due to multiple causes. Embolic occlusion of the proximal Artery of Percheron would be one of the differentials. The clinical signs would depend upon the anatomical areas of thalamus and or midbrain involved. Early imaging of the brain including MRI (DWI) may still not show the lesions. Common differentials are Wernicke’s encephalopathy, infection and demyelination. Recovery depends on the areas involved, some may recover completely. In our case the patient had both bilateral paramedian and midbrain involvement. He initially needed ventilatory assistance, and had a prolonged stay in the hospital. At discharge he was significantly disabled needing constant supervision and support for his activities of daily living.
